# Understanding Synaptogenesis and Functional Connectome in *C. elegans* by Imaging Technology

**DOI:** 10.3389/fnsyn.2016.00018

**Published:** 2016-06-29

**Authors:** Jung-Hwa Hong, Mikyoung Park

**Affiliations:** ^1^Center for Functional Connectomics, Korea Institute of Science and TechnologySeoul, South Korea; ^2^Department of Life Sciences, Korea UniversitySeoul, South Korea; ^3^Department of Neuroscience, Korea University of Science and TechnologyDaejeon, South Korea

**Keywords:** presynaptic assembly, synaptic specificity, synaptogenesis, functional connectome, neural circuits, imaging, *C. elegans*

## Abstract

Formation of functional synapses is a fundamental process for establishing neural circuits and ultimately for expressing complex behavior. Extensive research has interrogated how such functional synapses are formed and how synapse formation contributes to the generation of neural circuitry and behavior. The nervous system of *Caenorhabditis elegans*, due to its relatively simple structure, the transparent body, and tractable genetic system, has been adapted as an excellent model to investigate synapses and the functional connectome. Advances in imaging technology together with the improvement of genetically encoded molecular tools enabled us to visualize synapses and neural circuits of the animal model, which provide insights into our understanding of molecules and their signaling pathways that mediate synapse formation and neuronal network modulation. Here, we review synaptogenesis in active zones and the mapping of local connectome in *C. elegans* nervous system whose understandings have been extended by the advances in imaging technology along with the genetic molecular tools.

## Introduction

One of the fundamental goals of neuroscience is to understand the generation of functional nervous system that underlies neural basis of behavior and cognition. Extensive research has attempted to interrogate the molecular and cellular mechanisms of synapse formation and functional neural circuit development. Ever since it was proposed by Sydney Brenner in the mid 1960's (Brenner, 1974), the nematodes *Caenorhabditis elegans* (*C. elegans*) has been considered as an ideal model organism to study synaptic development and neural circuitry. The organism has relatively simple nervous system, having 302 neurons and its neurochemistry and genetics are similar to those of mammals. Moreover, the complete structure and connectivity of *C. elegans* nervous system have been deciphered through genetic screens and reconstruction of electron micrographs (EM) of serial sections, which led to discovery of novel molecules important for development and maintenance of functional synaptic connectivity (White et al., 1986). *C. elegans* with its transparent body was the first animal in which the green fluorescent protein (GFP) was expressed (Chalfie et al., 1994). Combined with its stable expression of fluorescently tagged proteins (Mello et al., 1991; Frokjaer-Jensen et al., 2008), studies with *C. elegans* have made major contributions to our knowledge on neural development, axonal migration, and synapse formation. Recently, selective plane illumination microscopy (SPIM) techniques such as tiling light-sheet SPIM (TLS-SPIM) (Fu et al., 2016) and inverted SPIM (iSPIM) (Wu et al., 2011) have been developed and utilized to achieve high spatiotemporal resolution 3-dimensional live imaging of *C. elegans* embyos with no detectable phototoxicity, which could enable studies on synaptogenesis and axon guidance during embryogenesis in *C. elegans*. Another recent work adopting complementation-activated light microscopy (CALM) in which proteins are conjugated with non-fluorescent split-fluorescent proteins, which become to be fluorescent when complemented with synthetic peptides enabled single-molecule imaging with a precision of 30 nm within synapses in live worms (Zhan et al., 2014).

Rapid developments of advanced imaging technologies have expanded our understanding of the molecular and cellular basis of synaptogenesis with great depth, taking a huge step closer to revealing functional neural connectome. Here, we discuss on the synaptogenesis in presynaptic active zones revealed by both conventional and advanced imaging set-ups and review recent work utilizing advanced imaging technology to unravel the functional connectome of neural circuits. Rather than dealing with the mechanistic aspects of synapse formation and neural circuits development, this review will mainly focus on how synaptic ultrastructure, synaptic formation, and functional neural connectome have been sophisticated by the advanced imaging technology. For more in-depth reviews on the mechanism of synaptogenesis, synaptic specificity, and neural circuits development, see Campbell et al. (2015), Cherra and Jin (2015), Jin (2015), Zhen and Samuel (2015), Yogev and Shen (2014), Chia et al. (2013), and Park and Shen (2012).

## Imaging synapse assembly

Chemical synapses are specialized intercellular junctions with two apposed compartments, the pre-synaptic terminal and the postsynaptic target, and the synaptic cleft which is about 20 nm gap between the pre- and postsynapses (Cowan and Kandel, 2001). Proper organization of pre- and postsynaptic components with precise regulation underlies formation of functional synapses. For the past decades, tremendous details regarding the morphology and assembly of *C. elegans* synaptic structure have been revealed with development of genetic tools and imaging technology. This section focuses on presynaptic assembly and synaptic specificity revealed by genetically encoded molecular tools and imaging technologies.

### Presynaptic active zone imaging

The presynaptic compartment in *C. elegans* exhibits an overall structural organization similar to that in vertebrates, with synaptic vesicles clustered in and around the electron-dense membrane structure called active zone known to serve as a major site of neurotransmitter release. Ultrastructural analysis have shown that, despite the variations among the appearances, synapses of various organisms commonly display synaptic vesicle docking and fusion at active zone that can be identified by darkly stained membrane structures (Zhai and Bellen, 2004; Ackermann et al., 2015).

Many studies using *C. elegans* have investigated the role of various proteins localized at active zone in synapse formation (Yeh et al., 2005; Watanabe et al., 2011). Classical EM analysis has provided initial assessment of *C. elegans* synaptic components but its requirement for ultrathin sectioning of samples approximately 50 nm thickness (White et al., 1986) limits the resolution and impairs detailed visualization of fine structures. The multifunctional synaptic scaffolding protein SYD-2/liprin-α is one of the key proteins identified to regulate synaptic development in *C. elegans* and *Drosophila* (Zhen and Jin, 1999). The loss-of-function analysis on SYD-2/liprin-α and uncoordinated-10 (UNC-10)/Rab3-interacting molecule (RIM), which is another dense-projection components (Weimer et al., 2006) revealed reduced vesicle recruitment at active zone (Stigloher et al., 2011; Kittelmann et al., 2013), and smaller dense-projection due to loss of SYD-2/liprin-α function (Kittelmann et al., 2013) unlike the finding showing an expanded dense-projection (Zhen and Jin, 1999). One suggested explanation for variability in *syd-2* mutant synaptic ultrastructure is due to the differences in fixation procedure (Kittelmann et al., 2013). Nevertheless, it is certain that advanced and optimized imaging technique led to identification of regulatory proteins to retain synaptic vesicle at active zone.

A method which comprises of correlative fluorescence electron microscopy was developed and optimized to observe the nanoscopic localization of SYD-2/liprin-α in *C. elegans* active zone (Watanabe et al., 2011). The technique employed both stimulated emission depletion (STED) microscopy and photoactivated localization microscopy (PALM) on ultrathin sections for protein localization at super-resolution nanoscale level and subsequently correlate the protein localization with ultrastructures by electron microscope. The localization of SYD-2/liprin-α to the *C. elegans* presynaptic dense-projection observed by this technique (Watanabe et al., 2011) was consistent with the earlier finding from the immunoelectron micrograph (Yeh et al., 2005) but the result was more advanced to provide the precise localization of the proteins in small and dense structures likely within the synapse at the level of nanoscale super-resolution.

In addition, studies using advanced EM tomography of 250 nm thick sections combined with high-pressure freezing (HPF) and freeze substitution (Stigloher et al., 2011; Kittelmann et al., 2013) have resolved the highly complex structure of dense-projections at cholinergic neuromuscular junctions (NMJs) of *C. elegans*, revealing composition of building units forming bay-like structures in which synaptic vesicles are docked to the active zone membrane. Furthermore, serial reconstruction of HPF EM sections and EM tomography enabled the construction of a high-resolution 3D model of presynaptic ultrastructure, overcoming resolution limitation raised by the conventional EM and revealing a physical link between dense-projections and synaptic vesicles within *C. elegans* presynaptic active zone.

### Presynaptic assembly imaging

**Figure 1:**
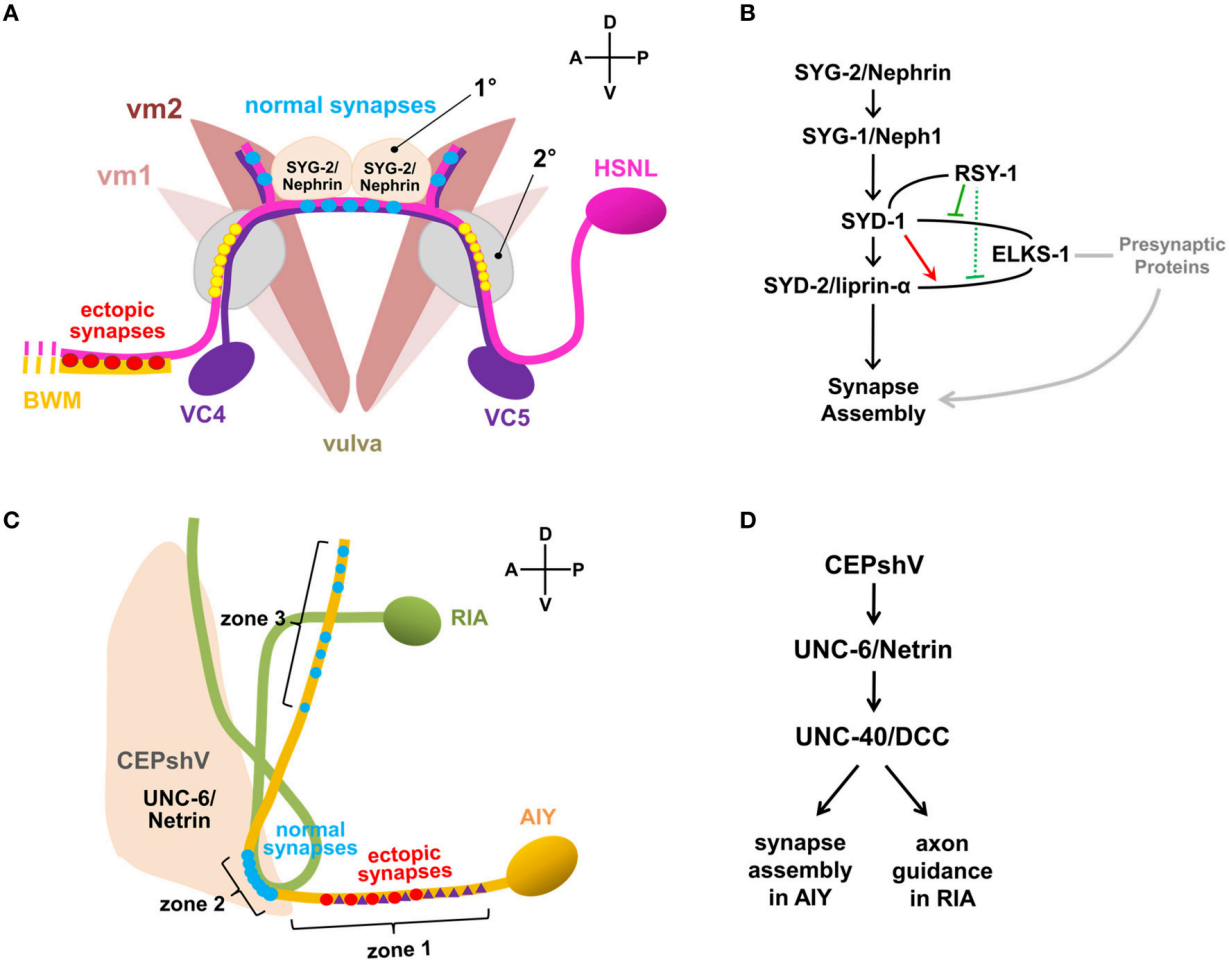
**Synaptic specificity regulated by non-neuronal factors. (A)** Synaptic connectivity of neurons and muscles associated in the egg-laying circuit of *C. elegans*. HSNL forms synapses with vulval muscle 2 (vm2) and ventral cord (VC) motor neurons, VC4 and VC5 specifically to the regions immediately adjacent to the primary epithelial cells (1°) which secretes SYG-2/Nephrin. Mutations in SYG-1/Neph1 or SYG-2/Nephrin disrupt synaptic specificity of HSNL and cause ectopic synapse formation with select body wall muscle (BWM). Ectopic positioning of SYG-2/Nephrin to the secondary epithelial cells (2°) recruited SYG-1/Neph1 to HSNL near the secondary epithelial cells, which was shown to be sufficient to form synapses ectopically at the sites where SYG-1/Neph1 is recruited (yellow circles). **(B)** Pathway for HSNL synapse assembly. SYG-2/Nephrin ensures proper localization of SYG-1/Neph1 which defines presynaptic localization of the active zone proteins. ELKS-1/ERC/CAST could function redundantly with SYD-1 or other unidentified presynaptic proteins that positively regulate synapse assembly (gray lines). In the presence of SYD-1, the SYD-2/liprin-α and ELKS-1/ERC/CAST interaction was enhanced (red arrow). In the presence of RSY-1, SYD-1, and ELKS-1/ERC/CAST interaction is weakened (solid green), suggesting RSY-1 as a negative regulator in the HSNL presynaptic assembly process likely by weakening the SYD-2/liprin-α and ELKS-1/ERC/CAST interaction (dotted green) indirectly through the RSY-1 and SYD-1 interaction. Plain lines indicate biochemical interactions. **(C)** Synaptic connectivity of AIY and RIA interneurons regulated by ventral cephalic sheath cells (CEPshV) at *C. elegans* nerve ring. Synapses between AIY and RIA are formed *en passent* as they are ensheathed in zone 2 by CEPshV, which secretes UNC-6/Netrin that regulates UNC-40/DCC activity in AIY. Abnormal distend positioning of CEPshV toward zone 1 causes ectopic localizations of both presynapses (red circles) and UNC-40/DCC (purple triangles) in zone 1 of AIY. **(D)** Pathways for AIY and RIA connectivity. CEPshV secretes UNC-6/Netrin, which regulates both positioning of presynapses in AIY and axon guidance of postsynaptic RIA through UNC-40/DCC activity to the location specified by CEPshV.

Cell type-specific tagging of synaptic proteins with fluorescent reporter has been a key reagent to study synaptogenesis and its regulation in *C. elegans* (Nonet, 1999; Shen and Bargmann, 2003; Sieburth et al., 2005; Yeh et al., 2005). Hierarchical assembly of presynaptic active zone was observed in *C. elegans* HSNL synapses by fluorescently labeling the multiple active zone proteins and expressing them in the various mutant animals (Patel et al., 2006). Fluorescent protein fused with a synaptic vesicle-associated protein RAB-3 visualized synaptic vesicle clusters and confirmed the presynaptic localization of various active zone components, including SYD-1, SYD-2/liprin-α, ELKS-1/ERC/CAZ-associated structural protein (CAST), GIT, and SAD-1 kinase in the HSNL synapses (Patel et al., 2006). Altering the location of SYG-1/Neph1 by ectopically expressing SYG-2/Nephrin in the secondary vulval epithelial cells, led to ectopic localizations of presynaptic components, including RAB-3, SYD-1, SYD-2/liprin-α, GIT, and ELKS-1/ERC/CAST in the HSNL regions where the secondary vulval epithelial cells made contacts (Figure 1A). This supported the idea that along with SYG-2/Nephrin as an upstream signal of SYG-1/Neph1, SYG-1/Neph1 defines the presynaptic localization and is sufficient to recruit presynaptic components, including the two key scaffold molecules SYD-1 and SYD-2/liprin-α (Zhen and Jin, 1999) to the regions defined by its localization (Patel et al., 2006; Figure 1B).

In *syd-1* and *syd-2* mutants, the presynaptic components, including RAB-3, ELKS-1/ERC/CAST, GIT, SAD-1, UNC-57/endophilin, and SNN-1/synapsin-1 were failed to be assembled, identifying those presynaptic components as downstream molecules of SYD-1 and SYD-2/liprin-α in the active zone assembly process (Patel et al., 2006). Gain-of-function mutation (Dai et al., 2006) or overexpression of SYD-2/liprin-α (Patel et al., 2006) in *syd-1* mutants completely restored the synaptic accumulation of SNB-1/synaptobrevin, whereas the SYD-1 overexpression in *syd-2* mutants was not sufficient to induce the rescue effect (Patel et al., 2006), illustrating the SYD-1 and SYD-2/liprin-α mediated presynaptic assembly with the SYD-1 as an upstream of SYD-2/liprin-α (Figure 1B). Although the loss of ELKS-1 function by itself did not induce apparent defects in synapse assembly in *C. elegans* HSNL synapses (Dai et al., 2006; Patel et al., 2006), synapse formation in the *syd-2* gain-of-function and *syd-1* double mutants exhibited a high dependency on ELKS-1 expression (Dai et al., 2006), suggesting that ELKS-1 functions redundantly with SYD-1 or other presynaptic proteins that positively regulate synapse assembly (Figure 1B).

Regulator of synaptogenesis-1 (RSY-1) was cloned as a negative regulator of synapse formation for its deletion mutants to lead to extra synapse formation and exhibit increased accumulation of SNB-1/synaptobrevin at presynaptic sites in the HSNL (Patel and Shen, 2009). A single-cell *in situ* protein-protein interaction assay revealed enhanced interaction between SYD-2/liprin-α and ELKS-1/ERC/CAST in presence of SYD-1 while direct interaction between SYD-1 and ELKS-1/ERC/CAST is weakened in presence of RSY-1, suggesting RSY-1 as a negative regulator of *C. elegans* HSNL synapse assembly likely by weakening the SYD-2/liprin-α and ELKS-1/ERC/CAST interaction indirectly through its interaction with SYD-1 (Figure 1B). Together, presynaptic differentiation at *C. elegans* HSNL synapses was initiated by SYG-1/Neph1, a synaptic specificity molecule that defines the location of presynaptic sites along the HSNL axon, leading to activate the presynaptic assembly process by recruiting the two key scaffolding proteins SYD-1 and SYD-2/liprin-α. SYD-2/liprin-α-centered assembly of presynaptic components was achieved through the inter-communications among positive (SYD-1 and ELKS-1/ERC/CAST) and negative (RSY-1) regulators (Figure 1B).

## Imaging synaptic specificity

During the event of synapse formation, a precise apposition between the presynaptic release sites and postsynaptic receptors must be accomplished to ensure a rapid neurotransmitter release and reliable synaptic response. Neurons can select subpopulations of neurons they form synapses onto and can also select the defined specific subcellular sites to establish synapses (Akins and Biederer, 2006; White, 2007; Margeta and Shen, 2010). Such synaptic specificity is achieved by trans-synaptic adhesion between pre- and postsynaptic neurons (Yamagata et al., 2002; Graf et al., 2004; Choe et al., 2006), adhesion between the presynaptic neuron and a guidepost cell (Shen and Bargmann, 2003; Shen et al., 2004), molecules secreted from pre- or postsynaptic neurons (Umemori et al., 2004; Inaki et al., 2007) or from a guidepost cell (Christopherson et al., 2005; Colon-Ramos et al., 2007). In *C. elegans*, synaptic contacts are usually formed *en passant*, in which synapses are formed along the adjacent processes but not its terminus (White et al., 1986). Synaptic specificity studies in *C. elegans* have been accelerated upon the development of the expression tool of fluorescently tagged proteins in specific cell types driven by cell type-specific promoters, which enabled researchers to specifically label pre-, postsynapses, and neighboring guidepost cells (Nonet, 1999; Shen and Bargmann, 2003; Grunwald et al., 2004; Francis et al., 2005; Sieburth et al., 2005; Yeh et al., 2005; Hoerndli et al., 2013).

A specific synaptic connectivity between amphid interneuron Y (AIY) and ring interneuron A (RIA) in *C. elegans* nerve ring, considered as brain of the animal, was fluorescently visualized by expressing presynaptic RAB-3 in AIY and postsynaptic glutamate receptors GLR-1 in RIA (Colon-Ramos et al., 2007; Shao et al., 2013) (Figure 1C). The localization of synaptic connectivity between AIY and RIA has shown to be restricted in the zone 2 of AIY axon (Figure 1C) and such specificity is achieved by activation of both UNC-6/Netrin, a well-known axon guidance molecule that is exclusively expressed by glia-like ventral cephalic sheath cells (CEPshV) (Wadsworth et al., 1996) and the netrin receptor UNC-40/Deleted in Colorectal Cancer (DCC) (Colon-Ramos et al., 2007), supporting the idea that secreted molecules from glia govern synaptic specificity. Confocal microscopy revealed the projection of the CEPshV processes with respect to the region of innervation between AIY and RIA (Figure 1C). Loss-of-function in either UNC-34/enabled, a regulator of the actin cytoskeleton (Colon-Ramos et al., 2007) or circuit maintenance abnormal protein (CIMA-1), a regulator of synaptic maintenance in *C. elegans* (Shao et al., 2013), caused morphological alterations in CEPshV which migrated toward further posteriorly to ensheath AIY axon in zone 1 (Figure 1C). Morphological alterations in CEPshV led to ectopic localization of both UNC-40/DCC and presynaptic components in zone 1 (Figure 1C) due to the existence of UNC-6/Netrin secreted from CEPshV in zone 1 (Colon-Ramos et al., 2007). The process of RIA in *unc-34* mutants also abnormally migrated toward zone 1 where the ectopic synapses were formed (Figure 1C). Together, UNC-40/DCC plays two independent roles in each neuron, which are positioning of presynapses in AIY and axon guidance of postsynaptic RIA to the location specified by CEPshV (Figure 1D). These findings further support the model of non-neuronal contribution to the regulation of precise localization of synaptogenesis.

Earlier than the AIY-RIA synaptic specificity study, the *C. elegans* egg-laying circuit, which is predominantly innervated by the two hermaphrodite-specific motor neurons (HSNs), HSNL and HSNR, and the two ventral cord (VC) motor neurons, VC4 and VC5 has been reported to be regulated by non-neuronal factor. HSNL and HSNR synapse onto vulval muscle cells and onto the VC4 and VC5 neurons, while VC4 and VC5 neurons also synapse onto the vulval muscle cells. Despite the direct contact between HSN and VC processes, synapses formed between these cells are only restricted to the regions adjacent to the vulva (White et al., 1986) (Figure 1A). The specific positioning of synapses and the recognition between HSNL and its target were determined by adjacent vulva epithelial guidepost cells that express SYG-2/Nephrin. SYG-2/Nephrin interacts with SYG-1/Neph1 expressed in the HSNL, to recruit SYG-1/Neph1 to the site along the HSNL axon where presynaptic sites are developed (Shen and Bargmann, 2003; Shen et al., 2004) (Figure 1A).

More recently, introduction of the GFP reconstitution across synaptic partners (GRASP) developed in *C. elegans* has overcome the challenges addressed by labor-intensive conventional EM analysis and increased the spatial resolution to visualize the pre- and postsynaptic contacts. GRASP is based on functional complementation between two non-fluorescent split-GFP fragments separately expressed in the pre- and postsynaptic neurons, which label synapses between two cells of close proximity in living animals (Feinberg et al., 2008). Using GRASP, specific visualization of synaptic contacts between AIY and RIA was observed with high spatial resolution (Shao et al., 2013). In addition, GRASP revealed restricted synaptic localization between AIY and CEPshV (Shao et al., 2013), which is consistent with the published EM data (White et al., 1986). Formation of ectopic synapses between AIY and CEPshV due to morphological alteration in CEPshV was confirmed as well (Shao et al., 2013) (Figure 1C). GRASP application has also confirmed the SYG-1/Neph1 and SYG-2/Nephrin as synaptic specificity regulators of HSN synapses with vulval muscles and VC neurons. Analyzing GRASP fluorescence in wild-type and *syg-1* or *syg-2* mutants recapitulated the synaptic connectivity of HSN neurons (Feinberg et al., 2008) (Figure 1A). Besides the *C. elegans* nervous system, the GRASP has also been widely adapted by other model systems, such as *Drosophila* (Gordon and Scott, 2009; Gong et al., 2010) mouse (Kim et al., 2012; Yamagata and Sanes, 2012) and the cultured hippocampal neuronal system (Tsetsenis et al., 2014). Lately, newly modified GRASP strategies, involving activity-dependent synaptic GRASP and multi-color fluorescence reconstitution across synapses (X-RASP) have been validated in *Drosophila*, allowing preferential labeling of active synapses and multi-color labeling of active synapses in one animal (Macpherson et al., 2015; Li et al., 2016). Continuous development of GRASP shows the potential to expand the utility of GRASP to identify and map synaptic connectivity of neural circuits in the living animal with high resolution.

## Imaging functional neural circuits

An underlying goal of neuroscience is to understand the neural connectome that are responsible for synaptic function and neuronal basis of behavior. Anatomical structural connectome of the whole nervous system of *C. elegans*, which has been fully mapped by EM of serial sections (White et al., 1986), has served as a useful resource for researchers to study circuit function, thus making the *C. elegans* nervous system as an excellent model to investigate functional connectome of neural circuits. For the past decade, optogenetics has been widely adapted to manipulate neural circuits and examine the corresponding changes in synaptic function and behavior (Fang-Yen et al., 2012; Husson et al., 2013). Optogenetics uses genetically encoded light-sensitive proteins such as channelrhodopsins (Nagel et al., 2003, 2005), halorhodopsins (Han and Boyden, 2007; Zhang et al., 2007; Husson et al., 2012b), and archaerhodopsins (Ihara et al., 1999) as optogenetic actuators to either activate or inhibit neuronal activity via light and genetically encoded sensors such as GCaMP calcium indicator (Tian et al., 2009) and Clomeleon chloride indicator (Kuner and Augustine, 2000; Berglund et al., 2006) as optogenetic sensors to monitor responses to the synaptic inputs. This section will discuss various experimental imaging approaches to interrogate the neural connection using the *C. elegans* nervous system.

The initial optogenetics was applied to manipulate the behavior of *C. elegans* (Nagel et al., 2005). Expression of Channelrhodopsin-2 (ChR2), a blue light-gated depolarizing cation channel used to activate neural activity in *C. elegans* body muscles caused blue light-evoked contractions (Nagel et al., 2003, 2005), whereas expression of NpHR, a yellow light-gated hyperpolarizing chloride ion pump applied to inhibit neural activity in *C. elegans* muscle cells caused an extension of the worm's body length and locomotion defects by whole-field illumination of yellow-green light (Zhang et al., 2007; Husson et al., 2012b). Aside from NpHR, a yellow-green light-sensitive archaerhodopsin-3 (Arch) (Ihara et al., 1999) and a green-blue light-sensitive Mac (Waschuk et al., 2005) have also been expressed in *C. elegans* and induced a stronger optical silencing effect than NpHR likely due to efficient protein trafficking to the plasma membrane (Chow et al., 2010; Husson et al., 2012b). The simultaneous use of Arch and Mac enabled inhibition of two different neuronal subpopulations, depending on the illuminating lights used.

Light-sensitive probes expressed in *C. elegans in vivo* are mostly under the control of a promoter sequence. However, promoter-driven single cell expression of optogenetic protein is challenging to achieve due to the lack of single cell-specific promoter and instead proteins are diversely expressed, eliciting robust behavioral responses upon whole-field illumination (Husson et al., 2013). Although it may be useful for inspecting a novel optogenetic protein, optical manipulation of individual neurons needs to be accomplished in order to obtain insights into individual contribution by single neurons in functional connectivity. To this aim, new methods have been adapted in *C. elegans* to drive selective optical manipulation, either by genetically modulated single cell-specific expression of optogenetic protein (Ezcurra et al., 2011; Schmitt et al., 2012; Cho and Sternberg, 2014; Guo et al., 2015) or selective illumination of target neurons with a high spatial and temporal resolution (Guo et al., 2009; Leifer et al., 2011; Stirman et al., 2011; Husson et al., 2012a,b; Kocabas et al., 2012; Cohen et al., 2014; Luo et al., 2014; Shipley et al., 2014; Trojanowski et al., 2014) in order to dissect functional connections within the neural circuits (Table 1).

**Table 1:** **Cell-specific optogenetic applications in ***C. elegans*****.

**Single-cell stimulation approach**	**Circuit**	**Cells manipulated**	**References**
Cre or FLP recombinase application	Avoidance	ASH, AVA	Ezcurra et al., 2011
	Locomotion	ASH, AVA, PVC	Schmitt et al., 2012
	Avoidance circuit during sleep behavior	ASH, AVA, RIM, RIG	Cho and Sternberg, 2014
	Nociception and avoidance	ASH, ASI^*^, ADF^*^	Guo et al., 2015
Selective illumination	Locomotion	DB, VB	Leifer et al., 2011
	Avoidance	ASH, RIM	Guo et al., 2009
		ALM, AVM, PLM	Stirman et al., 2011
			Leifer et al., 2011
			Shipley et al., 2014
	Nociception	AQR, FLP, PVD	Husson et al., 2012a
		ASH, ALM, AVM	Husson et al., 2012b
		PVD	Cohen et al., 2014
	Chemotaxis	AIB, AIY, AIZ, RME, SMB	Kocabas et al., 2012
		ASER	Luo et al., 2014
	Feeding	MC, M1, M2, M4	Trojanowski et al., 2014

* *Optogenetic manipulation driven by neuronal type-specific promoters rather than Cre/FLP recombinase application*.

**Figure 2:**
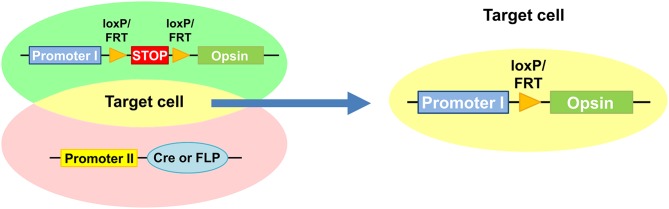
**Restricted expression of light-sensitive opsin mediated by Cre or FLP recombinases**. Promoter 1-containing construct is designed to drive expression of opsin with a fluorescent reporter. Promoter 2 drives expression of Cre or FLP recombinase. Conditional expression of opsin is mediated by the Cre or FLP recombinases by removing a transcription termination sequence flanked by loxP or FRT only in target cell where the both promoters are active.

Mainly adapted approach to specifically deliver light-sensitive opsins to individual neurons of *C. elegans* restricts the opsin expression by genetic application using Cre or FLP recombinases (Ezcurra et al., 2011; Schmitt et al., 2012; Cho and Sternberg, 2014; Guo et al., 2015) (Figure 2). The recombinase-dependent gene expression is driven by a set of two promoters, a first promoter driving the expression of opsin conjugated with a fluorophore along with or without a bicistronic fluorescent reporter and a second promoter driving the expression of Cre or FLP recombinase. In the first promoter-containing construct, a transcription termination sequence flanked by recombinase recognition sequences, loxP or FRT that are recognized by Cre or FLP recombinase is enclosed in front of opsin. The Cre or FLP recombinase-mediated recombination of loxP or FRT sites excised the stop sequence and allows conditional expression of opsin only in the target cell where both promoters are active (Husson et al., 2013) (Figure 2). Using Cre and FLP system, ChR2 were specifically expressed in PVC interneurons which evoked forward locomotion and in AVA interneuron and ASH sensory neurons which evoked backward-movement upon photostimulation (Ezcurra et al., 2011; Schmitt et al., 2012) (Table 1). Further effort to isolate exclusive expression of the light-sensitive proteins in a single cell (Ezcurra et al., 2011) would need to define the role of individual single neurons in functional neural circuits.

Instead of using genetically generated system and whole-field illumination, spatiotemporally patterned illumination of neurons expressing light-sensitive optogenetic proteins in immobilized *C. elegans* was used by for the first time *in vivo* using a digital micromirror device (DMD) whose individual mirrors can be controlled independently to precisely determine the location and size of the regions to be illuminated while simultaneously recording the calcium levels using a genetically encoded calcium sensor, GCaMP to analyze the functional connections among neurons. Combining the optogenetic actuator ChR2 and the sensor GCaMP with the patterned illumination via a DMD technology, the functional connections from the sensory neuron ASH to the interneurons AVA and AVD and the connections between the interneurons RIM and AVA have been monitored (Guo et al., 2009; Table 1).

Improvement in microscopic analysis and optogenetic illumination system allowed manipulation of neural activity in a freely behaving *C. elegans* with a high spatiotemporal resolution, providing an in-depth analysis on functional neural circuits underlying behavior at a single-cell level. A modified three-panel liquid crystal display (3-LCD) projector for simultaneous multicolor illumination and a motorized X-Y stage for keeping the unrestrained worm centered in the camera's field of view with a standard inverted epifluorescence microscope were systemized (Stirman et al., 2011; Husson et al., 2012b) and the Colbert system was equipped to control locomotion and behavior in real time (Leifer et al., 2011; Luo et al., 2014; Shipley et al., 2014). Spatial regulation of optical illumination is controlled either by estimating the coordinates of targeted cells using the machine-vision algorithms (Leifer et al., 2011; Trojanowski et al., 2014) or by calculating the anterior-posterior (A-P) axis (Stirman et al., 2011). Both systems have been instrumental in defining neural coding of several behaviors in *C. elegans* linked to the motor circuit, avoidance circuit, nociceptive circuit, chemotaxis circuit, and feeding circuits of freely moving worms (Table 1). Using AIY expressing ChR2 and targeted illumination by the DMD technology, it was shown that optogenetic manipulation of AIY activity alone was sufficient to evoke chemotactic behavior in freely moving *C. elegans*, and was suggested that AIY is plausible to act as a control node for coordinating other taxis behaviors as well (Kocabas et al., 2012). Another report using the Colbert system equipped with the DMD investigated an experience-dependent salt chemotaxis circuit. Optogenetic manipulation of neuronal activity of the ASER sensory neuron expressing ChR2 was shown to be connected to positive and negative chemotaxis in response to salt concentrations, indicating that ASER sensory neuron encodes the perception of salt concentration and the memory of the chemotactic set point in a chemotaxis circuit of *C. elegans* (Luo et al., 2014). In addition, optogenetic manipulations of specific pharyngeal neurons MC, M2, M4, and I1 in freely behaving worms by adopting ChR2 for optical stimulation and Mac for optical silencing along with the DMD for targeted illumination revealed a pharyngeal pumping/feeding circuit and identified the regulation of feeding rate by nicotinic and muscarinic receptors through the pharyngeal neuronal network (Trojanowski et al., 2014). Furthermore, multispectral illumination (Stirman et al., 2011) enables simultaneous application of optical stimulation and inhibition to an individual animal. Emerging studies have successfully facilitated multimodal optogenetic manipulation on *C. elegans* to independently excite different neurons in a single worm (Erbguth et al., 2012; Husson et al., 2012b; Schild and Glauser, 2015).

## Perspectives

*C. elegans* is currently the best organism to study synapses and neuronal circuits because the connectivity of its 302 neurons has been well-defined by serial reconstruction of EM (White et al., 1986), the body is transparent, and it is a genetically tractable animal model. *C. elegans* was one of the first organisms that GFP was expressed to label protein (Chalfie et al., 1994), GRASP was utilized to visualize specific synaptic contacts (Feinberg et al., 2008), optogenetics was applied to manipulate behavior of live animals (Nagel et al., 2005), and more recently, sonogenetics using low-pressure ultrasound was challenged to activate specific ultrasonically sensitized neurons and modify locomotory behavior (Ibsen et al., 2015).

As genetically encoded fluorescent proteins have been rapidly developed for the past decades since the GFP was introduced in the field, it is also expected that the number of optogenetic tools will rapidly increase to likely provide optogenetic proteins with different spectral properties (Zhang et al., 2008; Gradinaru et al., 2010) and ionic specificities (Han and Boyden, 2007; Zhang et al., 2007) and help expand the understanding of synaptic function and neural circuits. During such processes, it is confidently predicted that *C. elegans* will provide a systematic *in vivo* platform to test the optogenetic tools newly developed and to ultimately apply to the synaptic function and functional connectome studies. Together with the improvement of fluorescent and optogenetic tools, continuous development in *C. elegans* imaging technology will promise a breakthrough in deciphering functional neural connectome.

In addition to the monitoring and controlling of existing neuronal circuits via optogenetic applications and advanced microscopy systems as described in this review, it is very plausible to develop the ways to actively manipulate neural circuits for instance by inserting new connections or removing existing connections, resulting in the reprogramming of neural circuits. Indeed, a recent study on artificial modifications of neural circuits was reported in *C. elegans* by expressing transgenically targeted heterologous connexin to insert a new electrical synapse between normally unconnected neurons in intact animals, which resulted in altered salt taste and olfactory chemotaxis behavior (Rabinowitch et al., 2014). Conversely, laser ablation method can be used to remove existing connection (Sulston and White, 1980; Bargmann et al., 1993; McIntire et al., 1993; Fang-Yen et al., 2012; Rabinowitch et al., 2013). Such artificial modification of neural circuits not only help understand fundamental functions of neuronal connectivity underlying complex behavior but could also be applied to disease brain circuits with the purpose of therapeutics at the circuit level.

## Author contributions

All authors listed, have made substantial, direct and intellectual contribution to the work, and approved it for publication.

### Conflict of interest statement

The authors declare that the research was conducted in the absence of any commercial or financial relationships that could be construed as a potential conflict of interest.
